# Coronary stents with inducible VEGF/HGF-secreting UCB-MSCs reduced restenosis and increased re-endothelialization in a swine model

**DOI:** 10.1038/s12276-018-0143-9

**Published:** 2018-09-03

**Authors:** Hyun-Kyung Chang, Pyung-Hwan Kim, Dong Wook Kim, Hyun-Min Cho, Mi Jin Jeong, Dea Han Kim, Yoon Ki Joung, Kyung Seob Lim, Han Byul Kim, Han Cheol Lim, Dong Keun Han, Young Joon Hong, Je-Yoel Cho

**Affiliations:** 1 Department of Biochemistry, BK21 PLUS Program for Creative Veterinary Science Research and Research Institute for Veterinary Science, College of Veterinary Medicine, Seoul National University, Seoul, South Korea; 2 Department of Biomedical Laboratory Science, College of Medical Science, Konyang University, Daejeon, South Korea; 3 Center for Biomaterials, Korea Institute of Science and Technology, Seoul, South Korea; 4 Department of Biomedical Science, CHA University, Sungnam, South Korea; 5 Chonnam National University Hospital, Gwangju, South Korea; 6 Futuristic Animal Resource and Research Center, Korea Research Institute of Bioscience and Biotechnology, Ochang, Chungbuk, Korea

## Abstract

Atherosclerotic plaques within the vasculature may eventually lead to heart failure. Currently, cardiac stenting is the most effective and least invasive approach to treat this disease. However, in-stent restenosis is a complex chronic side effect of stenting treatment. This study used coronary stents coated with stem cells secreting angiogenic growth factors via an inducible genome-editing system to reduce stent restenosis and induce re-endothelialization within the artery. The characteristics of the cells and their adhesion properties on the stents were confirmed, and the stents were transplanted into a swine model to evaluate restenosis and the potential therapeutic use of stents with stem cells. Restenosis was evaluated using optical coherence tomography (OCT), microcomputed tomography (mCT) and angiography, and re-endothelialization was evaluated by immunostaining after cardiac stent treatment. Compared to a bare metal stent (BMS) or a parental umbilical cord blood-derived mesenchymal stem cell (UCB-MSC)-coated stent, the stents with stem cells capable of the controlled release of hepatocyte growth factor (HGF) and vascular endothelial growth factor (VEGF) successfully reduced restenosis within the stent and induced natural re-endothelialization. Furthermore, UCB-MSCs exhibited the ability to differentiate into endothelial cells in Matrigel, and HGF and VEGF improved this differentiation. Our study indicates that stents coated with UCB-MSCs secreting VEGF/HGF reduce the restenosis side effects of cardiac stenting with improved re-endothelialization.

## Heart disease: Coated stents reduce repeated blocking of arteries

Coating stents with modified stem cells may reduce the risk of repeated artery blocking in the treatment of heart disease. Currently, tubular stents are used to re-open arteries narrowed or blocked by the build-up of fatty deposits. However, if the artery is damaged by the stent or tries to reject it, the resulting inflammatory immune response can result in restenosis, or re-blocking. Je-Yoel Cho at Seoul National University, Republic of Korea, and co-workers trialed stents coated with modified stem cells in swine models. The stem cells are designed to secrete controlled levels of two growth factors, enabling them to differentiate into blood vessel lining cells and build a protective layer around the stent. In comparison with standard stents and stents coated with unmodified stem cells, the modified stents improved blood flow and reduced restenosis.

## Introduction

Coronary artery disease is an angiocardiopathy that severely impairs health, and it remains the principal cause of mortality worldwide. The goal of treatment is the restoration of blood flow in the clogged artery to a near-normal rate^1–3^. Coronary stents are a widely used treatment strategy to keep the arteries open. However, restenosis and stent thrombosis limit the success of stent treatment. Delayed or incomplete endothelial regeneration is a key factor of these events.

The endothelialization of coronary stents decreases in-stent restenosis^4–7^. This process is an important factor in thrombosis prevention and the reduction of vascular smooth muscle cells (VSMCs) proliferation and migration. Therefore, a coronary stent that is capable of rapid surface endothelialization may become a next-generation stent^7–10^. We used a very effective combination strategy of gene and cell therapies, in which genome-edited stem cells released proangiogenic growth factors, to improve re-endothelialization.

Vascular endothelial growth factor (VEGF) is one of the most effective signaling proteins that stimulates vasculogenesis^11,12^. Hepatocyte growth factor (HGF) is a pleiotrophic factor that induces motogenesis, mitogenesis, survival, and morphogenesis in some cell types^13–15^. Therefore, the integration of these genes into the genome of human umbilical cord blood-derived mesenchymal stem cells (U-Ms) enhances the ability of these cells to stimulate angiogenesis. We used the TALEN genome-editing system to integrate these genes into stem cells and introduce targeted double-strand breaks into the chromosome 19 safe-harbor site. We controlled gene expression with doxycycline using the Tet-on system. Our previous studies demonstrated that VEGF and HGF-secreting U-Ms (VEGF/U-Ms and HGF/U-Ms) enhanced angiogenesis in a rat myocardial infarction model and mouse hind limb ischemia model^13,16^. VEGF/U-Ms and HGF/U-Ms were very effective and powerful cell therapy systems for the restoration of blood vessels and blood flow.

Stents coated with polydopamine (pDA), fibronectin (FN), and extracellular matrix (ECM) enhance stem cell adhesion, including MSCs, to metallic stents^17^. An ECM is a biocompatible and cell-supporting substance that provides cells with mechanical and physiological support to increase cell survival, adhesion, proliferation, and differentiation^18,19^. The ECM also traps and holds some growth factors and soluble molecules via proteoglycans, which are its primary components^20^. However, the ECM requires a strong connector to attach to metal surfaces, and pDA and FN are used as chemical connectors. FN recognizes and binds to ECM molecules via integrin, and its carboxyl termini covalently bind to pDA. Therefore, the FN-pDA layers serve as linkers to immobilize ECM molecules, such as fibrin, collagen, heparin, and fibronectin, on the surface^21^. Dopamine is a strong adhesive molecule derived from the muscle^22^. Dopamine binds firmly to organic and inorganic surfaces via a catechol that consists of a benzene ring with two hydroxyl groups. pDA also provides a functional amine group to immobilize molecules on the surface. Stem cells may be efficiently seeded after coating the stents with these three components.

This study investigated the potential of stents seeded with angiogenic growth factor-secreting MSCs to enhance re-endothelialization and reduce restenosis via rapid re-endothelialization. We loaded coronary stents with functional stem cells (VEGF/U-Ms and HGF/U-Ms) and assessed the efficacies of in-stent stenosis reduction and coronary artery re-endothelialization in a swine model.

## Materials and methods

### Cell culture and cell preparation

UCB-MSCs (U-Ms) isolated from human umbilical cord blood (hUCB) were kindly provided by the Kang laboratory at Seoul National University. Cells were isolated from hUCB as previously described^23^. The Borame Institutional Review Board and Seoul National University approved the U-M isolation procedure (IRB No. 0603/001-002-07C1). The U-Ms were maintained in mesenchymal stem cell medium (KSB-3, Kangstem Biotech, South Korea) supplemented with KSB-3 and 10% fetal bovine serum (Rocky Mountain Biologicals Inc., MT, USA) at 37 °C in 5% CO_2_. Cells were transfected with NEON using the TALEN system and HGF- or VEGF-secreting plasmids as previously described^13,16^.

### Viability assay

Live cells on precoated stent material were imaged by fluorescence microscopy after labeling with green fluorescent dye (PKH67, Sigma, USA). The metal materials were coated with polydopamine, fibronectin, and ECM. Cell viability was tested using the crystal violet assay and cell counting. Cells (5 × 10^4^) were seeded onto stent material coated with fibronectin and an extracellular matrix layer and incubated for 7 days. A crystal violet solution (50 µl of 0.5%) was added, and an image of the stained cells was captured. Methanol (200 µl) was added to each well and incubated for 20 min at RT. The cell density was detected at 570 nm using a spectrophotometer (Epoch, BioTek, VT, USA). Cells were counted using a hemocytometer after trypan blue (Gibco, NY, USA) staining.

### Western blotting (WB)

WB was performed to confirm growth factor expression, as previously described^13,16^. Transfected cells (5 × 10^5^) were seeded onto the stent material for 24 h and treated with Dox (5 μg/ml) for 2 days. The conditioned medium was collected and precipitated using trichloroacetic acid (TCA, Sigma, USA). The pellet was dissolved in 200 µl of RIPA buffer (Thermo, IL, USA). SDS-PAGE was performed using a 1% acrylamide gel. HGF (R&D Systems, MN, USA) and VEGF (Cell Signaling, MA, USA) antibodies were used at a 1:1000 dilution.

### Conventional PCR and real-time quantitative PCR

Conventional PCR and real-time PCR were performed 7 days after cell seeding on the stent material to assess MSC marker expression. Supplementary Table 1 lists the primer sequences targeting CD166, CD105, CD90, CD45, CD14, and GAPDH. PCR was performed using the GoTaq polymerase (Promega, MN, USA) according to the following protocol: one cycle of 95 °C for 5 min, 35 cycles of 95 °C for 30 s, 60.1 °C for 30 s, 72 °C for 30 s, and a final cycle of 72 °C for 3 min. PCR products were evaluated using a 1.5% agarose gel. Real-time PCR was performed using the same PCR conditions and a SYBR Green-based method.

### Tube formation

Cells (5 × 10^4^ cells/well) were seeded onto a layer of BD Matrigel (BD Biosciences, CA, USA) in 24-well plates and exposed to high-glucose DMEM (HyClone, UT, USA) containing 2% FBS for 48 h. MSCs alone were used as a control. Cells were incubated for 12 h to allow the formation of tube-like structures. Tube formation was analyzed via counting the number of branches per high-power field.

### Stent preparation

The coating protocol and its effect on enhancing cell adhesion were published previously^17^. Briefly, an 18 mm × 1.8 mm cobalt–chromium stent was incubated with 1 mg/ml of dopamine (Sigma, BT, USA) dissolved in 10 mM Tris buffer (pH 8.5). The pDA-coated CoCr stent was washed three times using ultrasonication for 10 min. The pDA-coated CoCr stent was washed with deionized water and dried in air. The pDA-coated CoCr stent was placed in a 50-μg/ml FN solution at 37 °C overnight and washed for 10 min. NIH3T3 fibroblasts (ATCC, VA, USA), at a density 1 × 10^4^ cells/cm^2^, were cultured on the pDA-FN-coated CoCr stent for 10 days to form the ECM layer. Cells were decellularized using 20 mM NH_4_OH and 0.5% Triton X-100. The decellularized surface was washed with PBS and treated with 50 µg/ml of RNase A and 50 units/ml of DNase I for 24 h at 37 °C. HGF or VEGF/U-Ms (2.4 × 10^6^ cells) were seeded on the pDA-FN-ECM-coated stent.

### Confirmation of cell adhesion

The stents were coated with fibronectin and extracellular matrix, as previously described^10^. Cells (2.4 × 10^6^) were seeded on the stents and incubated for 12 h in a CO_2_ incubator. Cells on the stent were detected using fluorescence microscopy (July, NanoEntek, South Korea). Scanning electron microscopy (SEM) analysis was performed to assess the cells remaining on the stent after transplantation. The samples were rinsed with 2.5% glutaraldehyde in α-MEM without serum and fixed for 30 min at RT. The samples were fixed in 2.5% glutaraldehyde in 0.1 M Na-cacodylate (pH 7.2) with 0.1 M sucrose for an additional 30 min at RT. The samples were treated with 1% osmium tetroxide in distilled water for 1 h, followed by dehydration through a graded series of ethanol solutions from 70%, 80%, 95% to 100%. A freeze-dryer was used to dry the samples. Samples were mounted on aluminum holders and coated with a 10-nm conducting layer of gold platinum. Samples were examined in an SEM (Jeol JSM7400F, Tokyo, Japan) using a voltage of 10 kV.

### Transplantation into the swine model

The Ethics Committee of Chonnam National University Medical School and Chonnam National University Hospital approved this animal study (CNU IACUC-H-2013-12), which conformed to the Guide for the Care and Use of Laboratory Animals published by the US National Institutes of Health (NIH Publication No. 85–23, revised 1996). Yorkshire × Landrace F1 crossbred castrated male pigs (20–25 kg) were observed in the laboratory animal center of Chonnam National University Medical Institute for 5–10 days prior to the experiment.

Pigs were anesthetized with zolazepam and tiletamine (2.5 mg/kg, Zoletil50^®^, Virvac, Caros, France), xylazine (3 mg/kg, Rompun^®^, Bayer AG, Leverkusen, Germany), and azaperone (6 mg/kg, Stresnil^®^, Janssen-Cilag, Neuss, Germany). An intravenous (IV) catheter was placed in the marginal ear vein for the administration of fluids and emergency drugs, such as epinephrine and antiarrhythmic agents (amiodarone hydrochloride). IV fluid administration of 0.9% saline was continued throughout the experiment. Pigs were intubated, and anesthesia was maintained using an inhalation anesthetic of sevoflurane (1%) in oxygen (100%). The pigs were mechanically ventilated. Tramadol HCl (5 mg/kg, Trodon^®^, Aju Pharm, South Korea) was administered IV pre- and postoperatively to reduce pain. The stent was inserted into the coronary artery of an 8-week-old pig, and its placement was confirmed using angiography. We used the left anterior descending (LAD) and left circumflex (LCX) arteries. The pigs were premedicated with 100 mg of aspirin and 75 mg of clopidogrel daily for 5 days prior to the procedure. The pigs were also treated with the immunosuppressant cyclosporine (CIPOL.N, Chong Kun Dang, South Korea) for 3 days after stent transplantation.

### Stenosis imaging: angiography, OCT, and mCT

Imaging analyses for stenosis evaluation were performed as previously described^24^. Briefly, a follow-up coronary angiogram was performed 4 weeks post-stent transplantation. Pigs were anesthetized and sacrificed using an overdose of potassium chloride at the end of the experiment. Hearts were rapidly removed, extracted and grossly sectioned at 1-cm intervals. Myocardial sections were stained with a 2,3,5-triphenyl tetrazolium chloride (TTC) solution (1% in phosphate-buffered saline) for 30 min at 37 °C. Sectioned heart tissues were fixed in 10% neutral buffered formalin overnight and embedded in paraffin for histological analyses.

### Angiography

A 7F coronary artery-guiding catheter was placed within the opening of the coronary artery, and a baseline coronary angiogram was obtained using the nonionic contrast agent Omnihexol (Omnihexol 300, Korea United Pharm Co., Seoul, Korea) under fluoroscopic guidance and a mobile fluoroscopy system (BV Pulsera, Philips Medical Systems, Andover, MA, USA). Angiography was performed to confirm an obstruction of the mid-LAD. The guide wire, balloon catheter, and guiding catheter were removed, and the left carotid artery was ligated.

### Optical coherence tomography (OCT)

The carotid artery was excised, and the neointima of pig blood vessels was measured using OCT (Model C7Xr OCT Imaging System). A guide wire was connected to a water box dedicated to in vitro experiments, and the coronary artery was fixed to the guide wire. An imaging catheter (C7 Dragonfly) was inserted through the guide wire into the coronary artery. OCT images were obtained via connecting the imaging catheter and the Dragonfly Duo, and neointimal vessels were measured using LightLab imaging (offline review workstation). We calculated the neointimal area by subtracting the lumen area from stent area (Neointimal area = stent area − lumen area). Supplementary Table 2 presents the detailed analysis data.

### Microcomputed tomography (mCT)

Tomograms of each sample were acquired using a microcomputed tomography scanner (SkyScan 1172, MA, Bruker). Harvested coronary artery tissues were stored in a 10% formalin solution and transferred to deionized water prior to mCT analysis. Tissue was fixed vertically in a cylindrical plastic container and mounted on a specimen stub using soft clay. The plastic container was sealed using paraffin film to prevent sample drying. Scanning was operated at 10-W (100 kV/100 µA) X-ray generation power using an aluminum-copper filter. Images were recorded at a 0.4° rotation for one step. Acquired images were reconstructed and visualized using software (NRecon/CTan, MA, Bruker). The in-stent restenosis area (ISR area) was calculated by subtracting the area of the lumen from the area within the stent strut.

### Immunohistochemical (IHC) staining

The tissues were harvested, fixed in 4% paraformaldehyde (Wako), embedded in paraffin, and cut into 5-μm-thick sections (Leica, Buffalo Grove, IL, USA). Immunohistochemistry was performed using anti-von Willebrand factor (vWF) (ab6994, Abcam, USA), anti-fibrin (Abcam, USA), anti-CD31 (ab108595, Abcam, USA) and anti-lamin A + C (Abcam, USA) antibodies at a 1:100 dilution and the appropriate secondary antibodies. Tissue samples were also stained with hematoxylin and eosin (H&E) and Masson’s trichrome. TTC, H&E, and Masson’s trichrome stains were performed to evaluate the infarcted area of the ventricle. An experienced cardiac pathologist performed histological evaluations of the infarcted myocardium.

### Tumor formation

BALB/c nude mice (Orient Bio, South Korea) were used to evaluate tumor formation in vivo. U-Ms and HGF- or VEGF-secreting U-Ms (5 × 10^6^) were injected into mice in 50 μl of serum-free DMEM and 50 μl of Matrigel (Corning, NY, USA). MDA-MB-231 breast cancer cells were used as a positive control. Mice were observed for 4 weeks, and tumor volume was measured weekly. Mice were sacrificed using CO_2_, and tumor formation and weights were analyzed.

### Statistical analysis

The data are expressed as the mean values ± standard errors. The data were analyzed using Prism, and a *t*-test or ANOVA was used to compare the data of different experimental groups. Differences were considered significant when the *p* values were <0.05.

## Results

### Retention of HGF/U-Ms and VEGF/U-Ms growth and markers on stent material

**Fig. 1:**
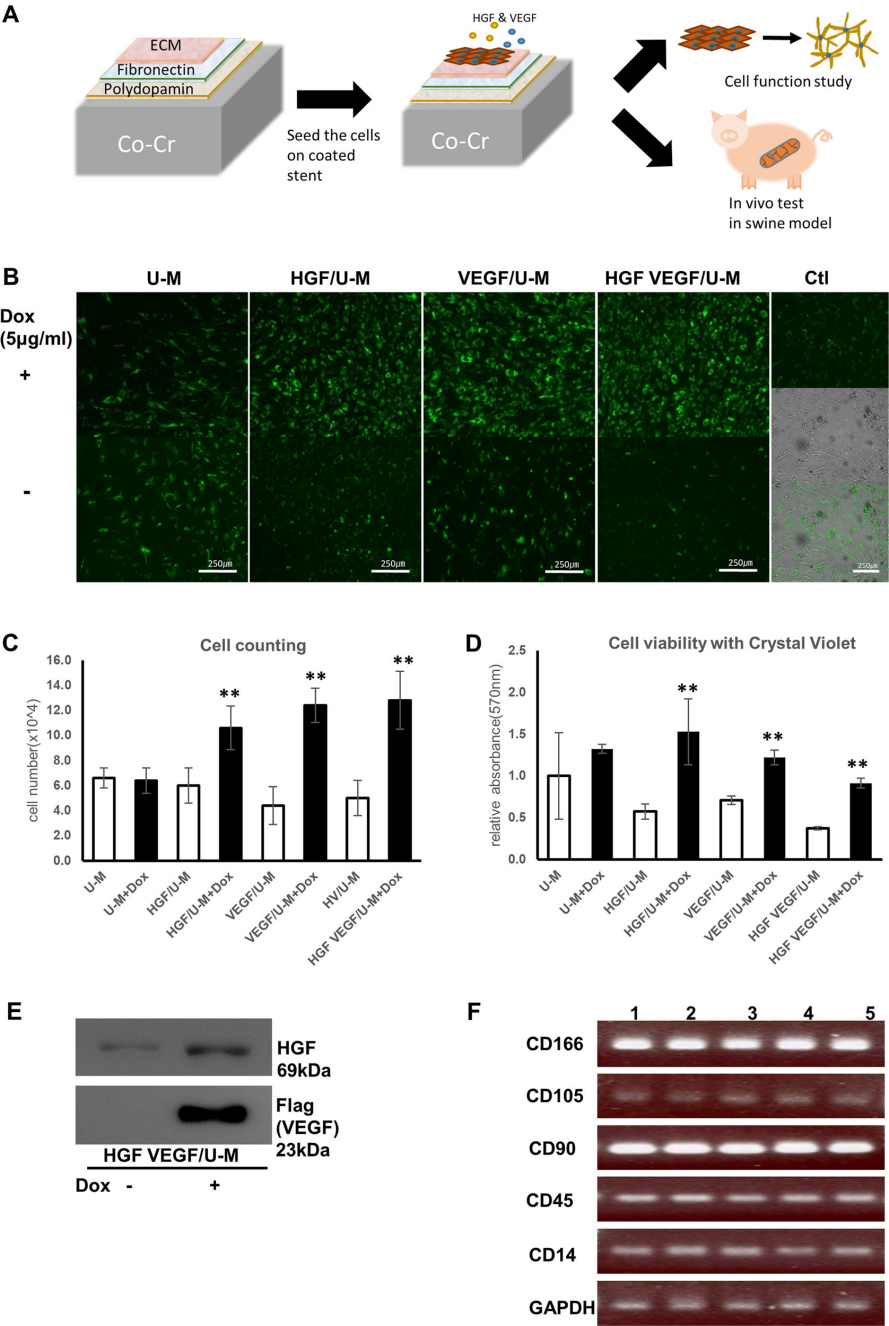
The properties of HGF/U-Ms and VEGF/U-Ms are maintained on stent material. **a** Schematic illustration of the experiment using the stent with HGF/U-Ms and VEGF/U-Ms to secrete the angiogenic factors in an inducible manner. The stents were coated with polydopamine, fibronectin, and extracellular matrix (ECM), followed by in vitro and in vivo swine experiments. **b** Human U-Ms secreting HGF and VEGF were seeded onto the pre-coated material sheets to confirm cell viability on the stent material. The cells stained with green fluorescent dye were detected using fluorescence microscopy. *U-M* human UCB-MSCs, *Ctl* control. *N* = 3. **c** The cells were counted at 7 days post seeding after detachment from the stent material and staining of dying cells using trypan blue (**denotes a *p*-value < 0.01). **d** Cell viability was analyzed using a crystal violet assay on the stent 7 days post seeding (** denotes a *p*-value < 0.01). **d**, **e** HGF and VEGF secretion were detected in conditioned media using western blotting. HGF/U-Ms and VEGF/U-Ms were treated with 5 µg of doxycycline for two days in a six-well plate. **f** MSC markers on the stent material were not altered, even in the HGF- and VEGF-secreting cells. Lane 1: U-Ms, 2: U-Ms + Dox, 3: U-Ms + Dox on material, 4: HGF/U-Ms + Dox on material, 5: HGF + VEGF/U-Ms + Dox on material. *N* = 3 experiments per group

The full experiment was performed as described in the schematic image (Fig. 1a). We examined whether the engineered cells were successfully implanted on the stent. We confirmed that the stent material did not affect cell growth or the expression of the UCB-MSC markers. Characteristics of the VEGF/U-Ms and HGF/U-Ms were evaluated after long-term interactions with the stent material. Cells were seeded on the materials for more than 7 days, and doxycycline (Dox) increased HGF- and VEGF-secreting U-Ms proliferation (Fig. 1b–d). HGF and VEGF secretion from the Tet-on system was confirmed using immunoblotting (Fig. 1e). HGF and VEGF secretion did not alter MSC markers (positive CD116, CD105, and CD90 markers and negative CD45 and CD14 markers) (Fig. 1f & sFig. 1A). We confirmed HGF and VEGF secretion and integration into the genome each time the cells were established on a stent (sFig. 1B). The effect of Dox on cell proliferation was a concern, but Dox did not affect MSC proliferation or morphology in this test (sFig. 2A & 2B). We confirmed that U-Ms maintained their properties and growth on the stent, and we demonstrated that HGF and VEGF secretion from the engineered stem cells promoted cell proliferation.

### Cells remained on the stents after coronary stent transplantation

**Fig. 2:**
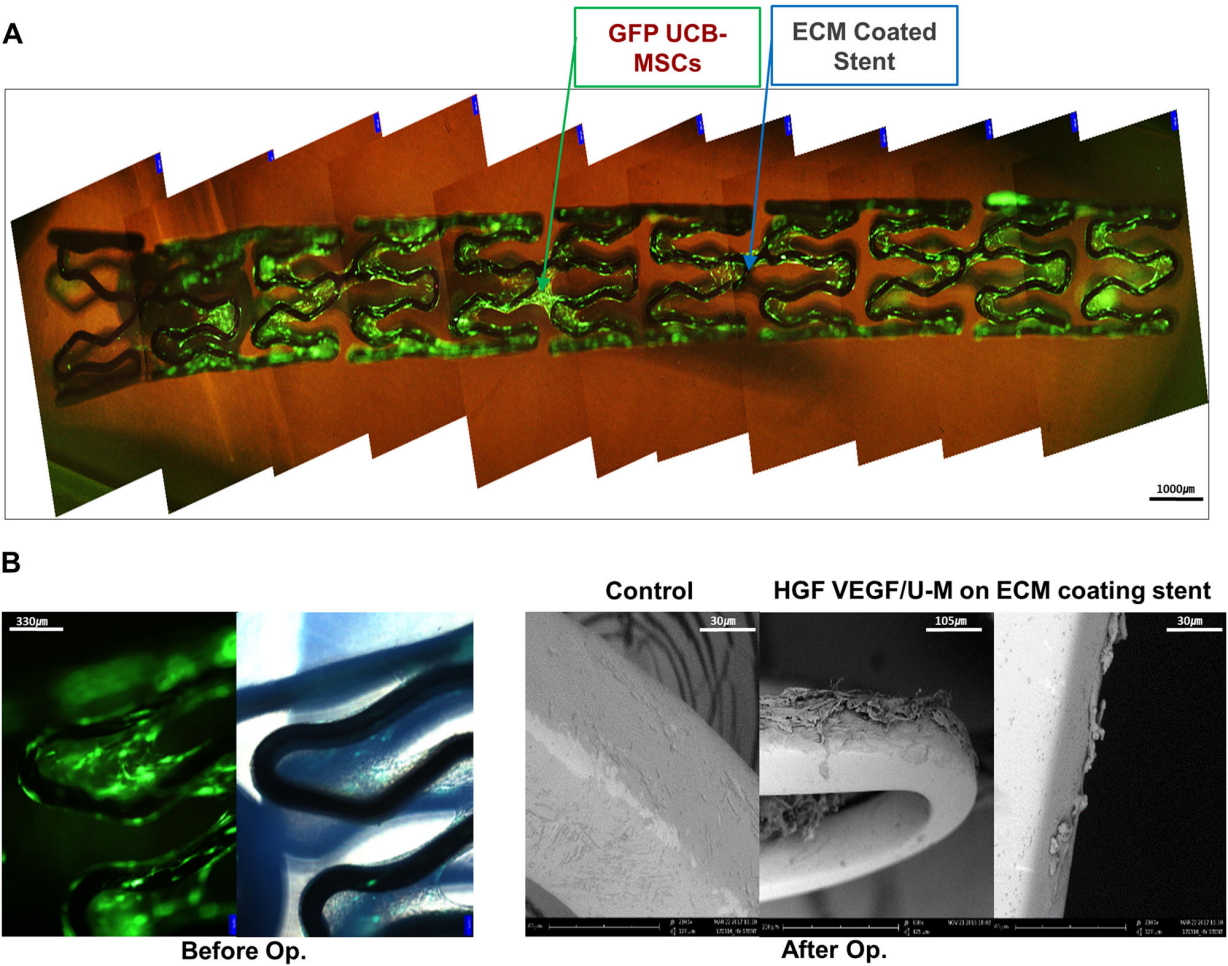
The cells remained on the stent after transplantation. **a** Fluorescent images showing adhesion of the GFP-expressing UCB-MSCs on the stents. **b** Fluorescent images prior to surgery and stent seeding in the swine model were captured using a microscope, and the SEM images after the end of the experiment were captured using scanning electron microscopy. *N* = 3

Cell adhesion to the bare stent material is limited; therefore, we designed stents with biocompatible matrices. Polydopamine and fibronectin were sequentially conjugated onto a CoCr stent. Fibroblasts were seeded onto stent surfaces and subsequently decellularized to provide the cell-secreted extracellular matrices (Fig. 1a). U-Ms with eGFP genes integrated in the Chr 19 safe-harbor site using TALEN were attached to the stent surfaces via the extracellular matrix. Cell adhesion was confirmed using fluorescence microscopy (Fig. 2a). Scanning electron microscopy (SEM) was performed to identify whether cells remained after mimicking stent implantation in the swine model due to concern about cell adherence on the stents after coronary artery transplantation. The surgical procedures led to some cell detachment due to the strong physical friction of balloon dilatation. However, cells remained on the lateral side of the stent after transplantation (Fig. 2b). These results suggest that the stem cells implanted on the stent remained after surgery.

### The effect of HGF and VEGF application periods on coronary restenosis

**Fig. 3:**
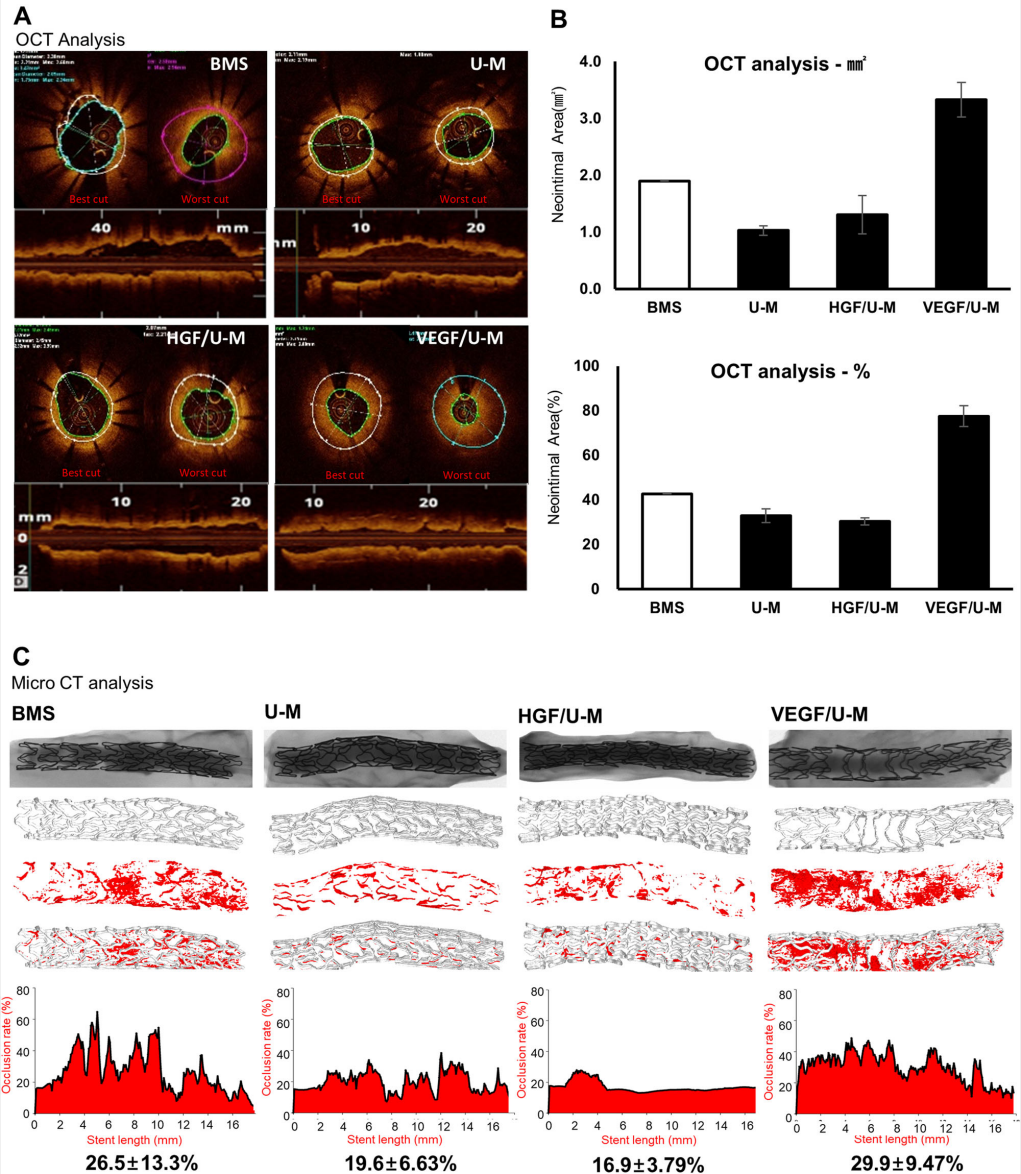
The single conditions of HGF/U-Ms and VEGF/U-Ms alone produced opposite effects on neointima levels 2 weeks after transplantation. **a** OCT images showing neointimal degrees 2 weeks after stent transplantation. *BMS* bare material stent, *U-M* human U-M-coated stent, *HGF/U-M* stent coated with HGF/U-Ms, *VEGF/U-M* stent coated with VEGF/U-Ms. **b** OCT analysis showing neointimal areas in mm^2^ and in % units. **c** The microCT results were analyzed at a 50 kV/200 μA spatial resolution with 17-μm units to show the surface smoothness and neointima areas. *N* = 3

Stents were applied to a swine model and observed for 2 weeks after stent implantation to evaluate the effects of the HGF/U-Ms- and VEGF/U-Ms-seeded stents on the coronary artery. The HGF/U-M group exhibited the lowest level of neointima formation among the bare metal stent (BMS) and stents with U-Ms, HGF/U-Ms, and VEGF/U-Ms (Fig. 3, HGF group). OCT and mCT analyses revealed that the HGF/U-M group exhibited the lowest amount of stenosis formation and a uniform pattern in the stenosis area (Fig. 3c). An even inner surface is a crucial/essential criterion for stent re-endothelialization. In contrast, excessive neointima formation was observed in the VEGF/U-M group after 2 weeks (Fig. 3, VEGF group). The VEGF group exhibited the lowest level of neointima formation in a 3-day trial (Supplemental Fig. 3), but this result was not observed at 2 weeks. The excessive amount of VEGF secreted from the VEGF/U-Ms may stimulate vascular smooth muscles during longer-term exposure to the cells. Other studies demonstrated that patients with increased VEGF after implantation exhibit a restenosis rate of 26.2% compared to that of 2.4% in patients with basal VEGF levels^25,26^. Restenosis was reduced in the HGF/U-M group, but this effect was not significant over the longer term. Therefore, the application of the cell population and the periodic conditioning of the HGF/U-Ms and VEGF/U-Ms must be optimized to promote re-endothelialization and minimize the neointima side effect.

### Optimization of HGF- and VEGF-secreting stem cells and enhanced tube formation with the HGF/U-Ms and VEGF/U-Ms

Stents with VEGF-secreting stem cells and longer implant times (2 weeks) produced more severe restenosis (Fig. 3). This effect may have occurred because VEGF is one of the strongest factors that stimulate the fibrosis and proliferation of all cells in the vascular area^26,27^. However, cells that only secreted HGF or VEGF did not efficiently reduce restenosis (Fig. 3). The combination of VEGF-A and HGF promoted neovascularization, especially re-endothelialization, under angiogenic conditions via enhanced intracellular signaling, which allows a more finely regulated control of the signaling molecules involved in the regulation of the cytoskeleton and cellular migration and morphogenesis^28^. Therefore, the combined condition is necessary, and the dose of HGF- and VEGF-secreting cells must be optimized. The amount of HGF and VEGF secreted from this system was analyzed in our previous studies. Fifty nanograms of VEGF and 2.2 ng of HGF were secreted from 2 × 10^6^ cells per day^13,16^. We maximized the HGF and minimized the VEGF based on these results because HGF promoted endothelial cells (ECs) more specifically, and VEGF increased overall vascular cell activity^29^. A 5:1 ratio of HGF- to VEGF-secreting cells was investigated in vitro, and the cell proliferation was similar to the control single condition (Fig. 1, HGF VEGF/U-M group). This condition included a range that is suitable in vivo^30^.

**Fig. 4:**
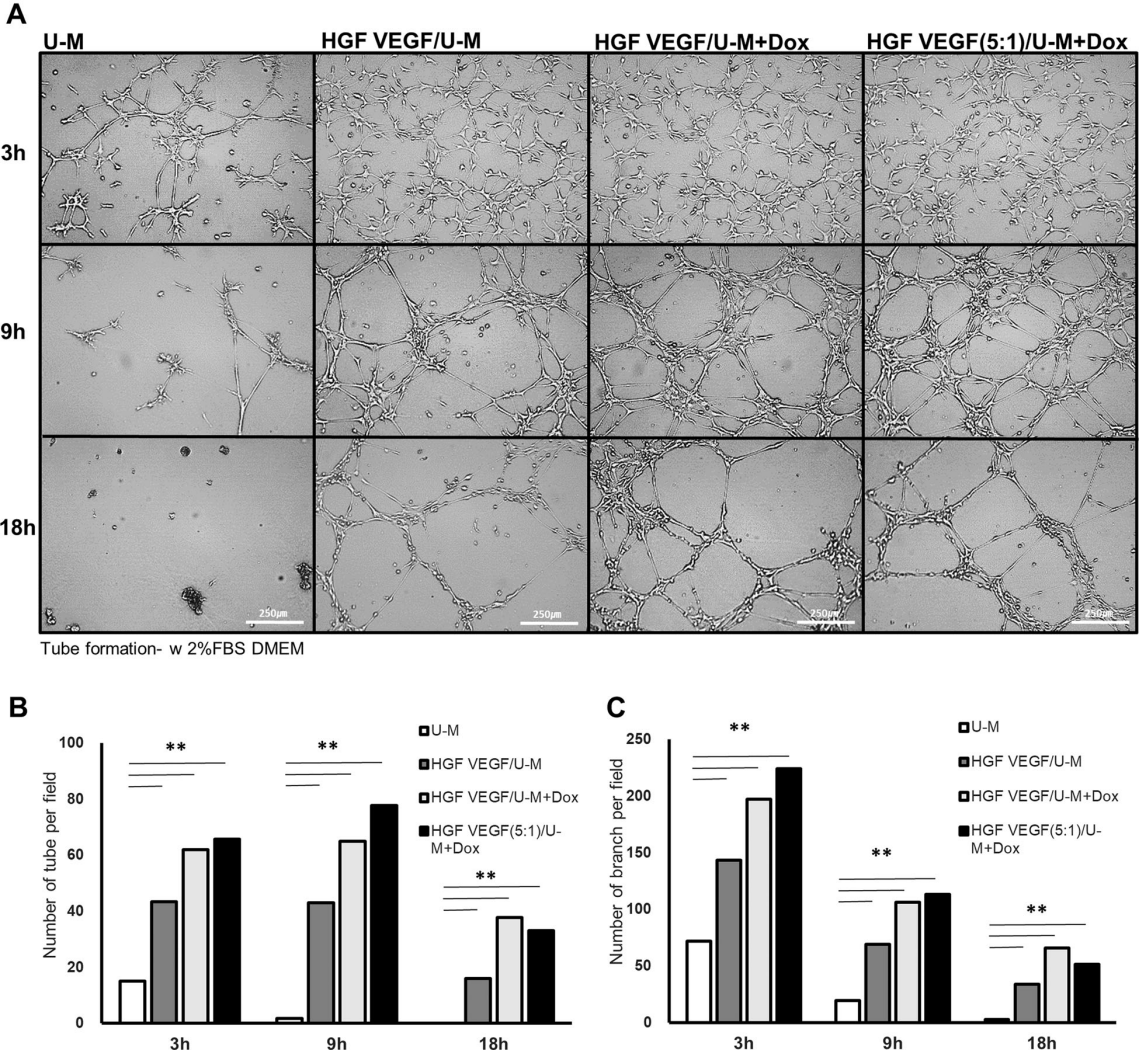
HGF + VEGF/U-Ms enhanced tube formation following HGF + VEGF induction. **a** Images of tube formation were acquired for the U-Ms (UCB-MSCs), HGF + VEGF/U-Ms (1:1 ratio) without doxycycline (Dox) and with Dox, and HGF + VEGF (5:1 ratio)/U-Ms with Dox. **b**, **c** Tube formation was analyzed based on the number of tubes (**b**) and the number of tube branches (**c**). *N* = 3

A source of endothelial cells is needed to facilitate coronary in-stent re-endothelization. Endothelial progenitor cells (EPCs), embryonic stem cells (ESCs) and induced pluripotent stem cells (iPSCs) are used as endothelial cell sources in vitro. Notably, MSCs differentiate into endothelial cells^31,32^, and we presumed that our functional HGF VEGF/U-Ms would differentiate into ECs. Tube formation using HGF + VEGF/U-Ms was observed on Matrigel, even under the culture condition that excluded endothelial differentiation medium and bFGF. However, the U-Ms did not form the tube properly in the absence of Matrigel (Fig. 4). HGF + VEGF secretion from cells in response to doxycycline induction (HGF + VEGF/U-Ms + Dox) enhanced the tube and branch numbers (Fig. 4b, c). The tubes persisted longer in the HGF + VEGF/U-Ms + Dox group (Fig. 4a). The 5:1 combination of HGF:VEGF/U-Ms in response to Dox stimulation resulted in better tube formation and branch numbers at early time points (3–9 h) (Fig. 4b, c). We examined HGF + VEGF-secreting stem cells at a 5:1 ratio in vivo for vascular re-endothelialization based on previous results and the tube formation results.

### Reduction of restenosis in the presence of HGF and VEGF (5:1)

**Fig. 5:**
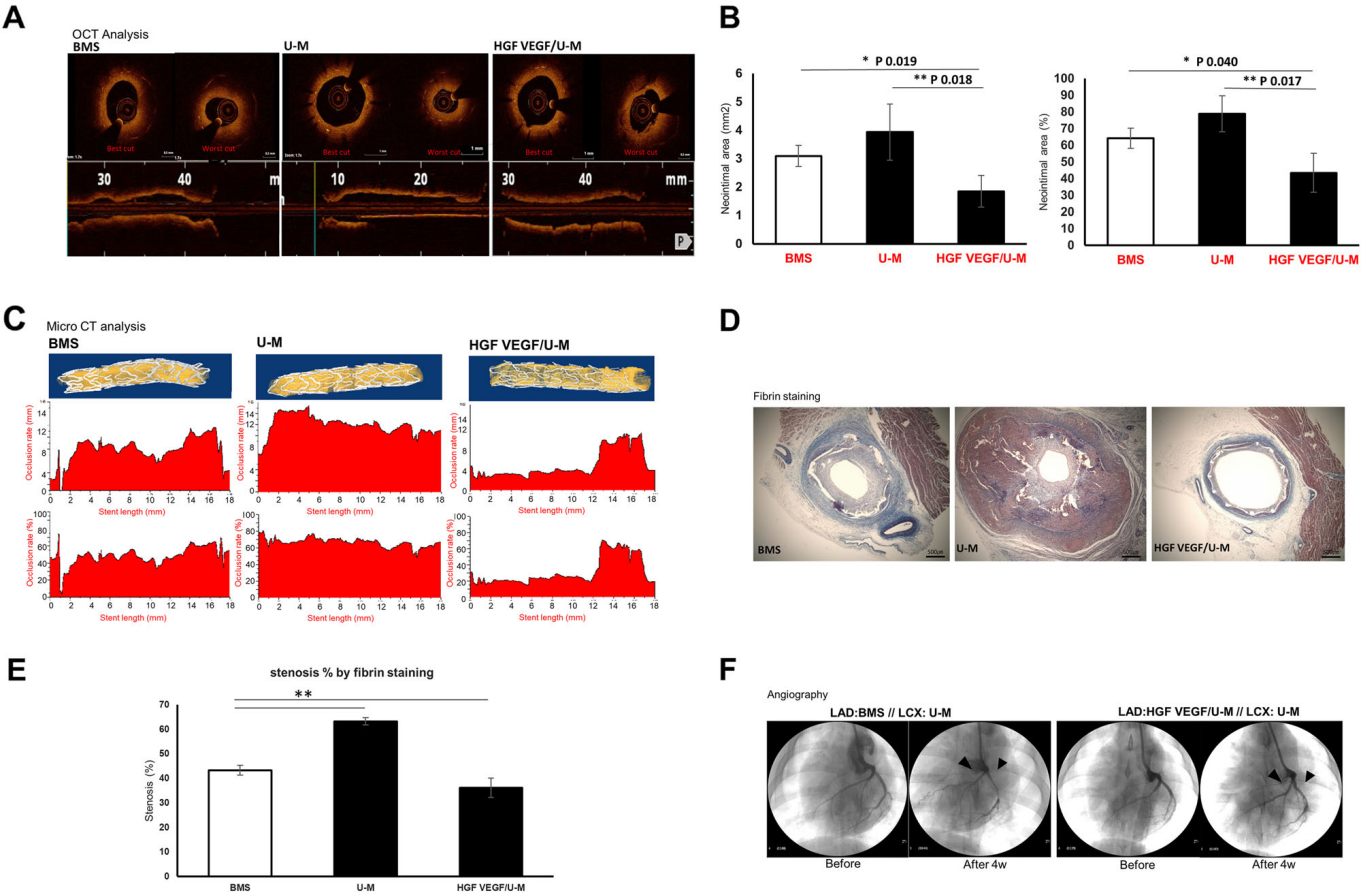
Stents coated with HGF + VEGF/U-Ms reduced the neointimal area 4 weeks after transplantation. **a** OCT results showing the neointima degree in the lumens of the coronary arteries 4 weeks after transplantation. **b** The neointima degree was analyzed in mm^2^ and the % of the neointima area (*; *p*-value of 0.019 and 0.040, **; *p*-value of 0.018 and 0.017). **c** Representative microCT images of the stents 4 weeks after transplantation. **d** Image of fibrin staining showing the fibrotic area with a cross-section of the transplanted stents. **e** The stenosis degrees were assessed as the % of fibrin staining (**p-value of 0.01). **f** Angiography showing the blood flow in the coronary artery with the transplanted stent. BMS; *N* = 6, U-M; *N* = 6, HV/U-M; *N* = 5

The HGF + VEGF/U-M combination stent was transplanted into the swine model to examine the efficacy of stents coated with HGF + VEGF (5:1)-secreting stem cells. Swine coronary arteries were observed 4 weeks after transplantation. The HGF/U-M + VEGF/U-M (+Dox) group exhibited the lowest restenosis compared to the BMS and U-M groups (Fig. 5). The HGF/U-M + VEGF/U-M group exhibited the lowest neointimal area in OCT (Fig. 5a, b) and mCT analyses (Fig. 5c). Fibrin staining also revealed the lowest fibrosis in the HGF/U-M + VEGF/U-M (+Dox) group (Fig. 5d, e). Live angiography analysis demonstrated normal blood flow in the HGF/U-M + VEGF/U-M (+Dox) group (Fig. 5f). Taken together, these results indicated that the swine coronary stents with HGF/U-Ms + VEGF/U-Ms in a 5:1 combination significantly decreased neointima formation and restenosis and provided the best blood flow.

### Enhanced re-endothelialization in stents with HGF/U-Ms+VEGF/U-Ms

**Fig. 6:**
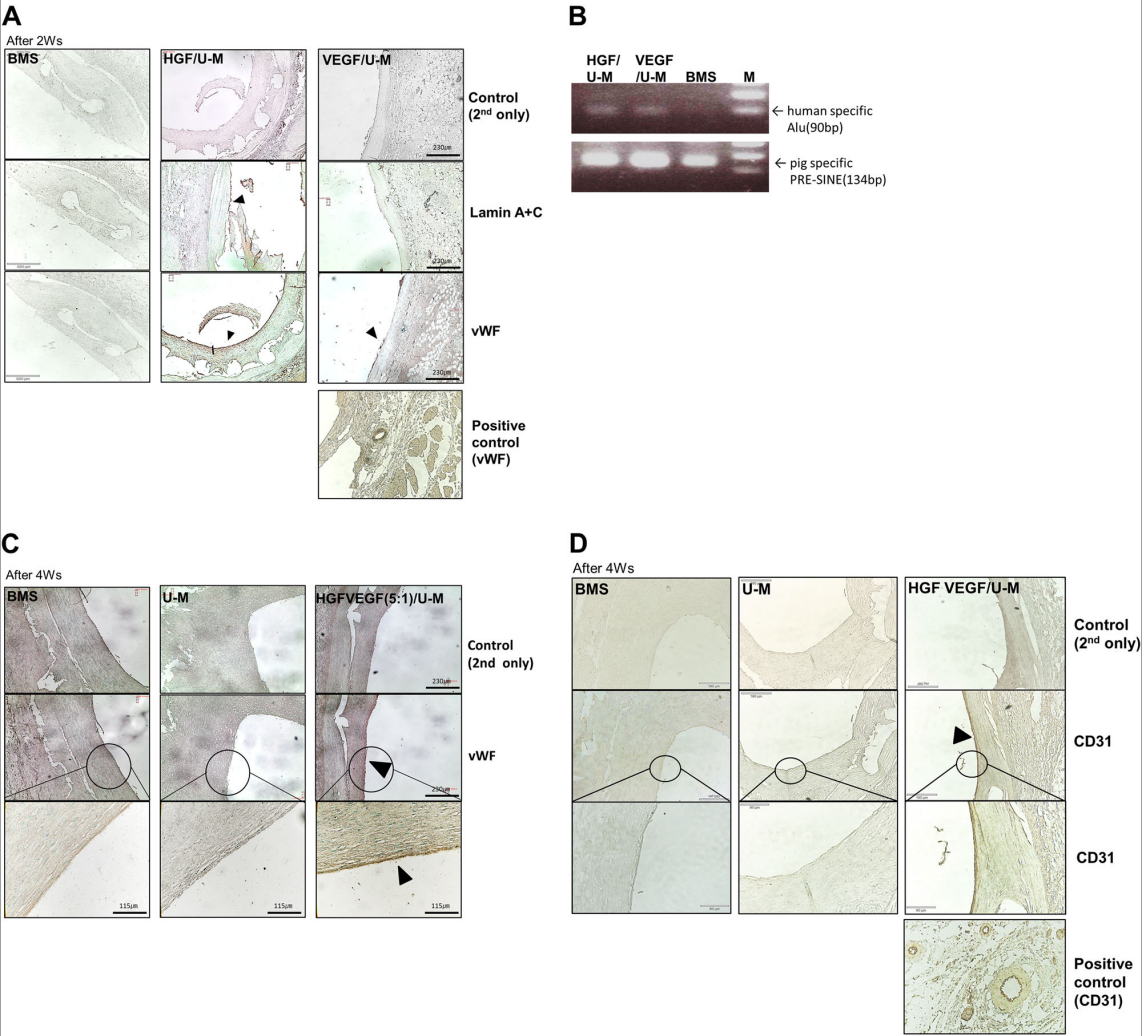
Re-endothelialization was observed in coronary arteries with transplanted stents coated with HGF + VEGF/U-Ms and human MSCs in the swine model. **a** The coronary arteries transplanted with stem cell-loaded stents were sectioned and stained using anti-vWF (1:200) and anti-Lamin A + C (1:200) antibodies. The samples were harvested 2 weeks after transplantation. **b** Human-specific Alu was detected using RT-PCR in coronary arteries 2 weeks after transplantation. **c** Anti-vWF immunostaining showing the re-endothelialized portion of the coronary arteries 4 weeks after transplantation for the stents coated with the HGF + VEGF/U-Ms. **d** Anti-CD31 (1:50) immunostaining showing the re-endothelialized region of the coronary arteries. *N* = 3

We stained the stent area of the coronary artery to visualize re-endothelialization after stent transplantation. Immunostaining for the endothelial cell markers vWF and CD31 revealed EC layers in the HGF/U-M and VEGF/U-M groups 2 weeks after transplantation (Fig. 6a). The HGF/U-M + VEGF/U-M (5:1) group exhibited vWF and CD31 staining 4 weeks after transplantation, which indicates re-endothelialization. No vWF and CD31 staining was observed in the MSC only or BMS groups (Fig. 6c, d). Transplanted human MSCs were observed in the HGF/U-M and VEGF/U-M groups using genomic PCR to detect human-specific Alu (Fig. 6b). Human-specific Alu was detected in the HGF/U-M and VEGF/U-M stent groups, but not in the BMS group. These results suggest that human cells coated the endothelium after HGF/U-M or VEGF/U-M transplantation in the stent.

### HGF/U-Ms+VEGF/U-Ms did not induce tumor formation

**Fig. 7:**
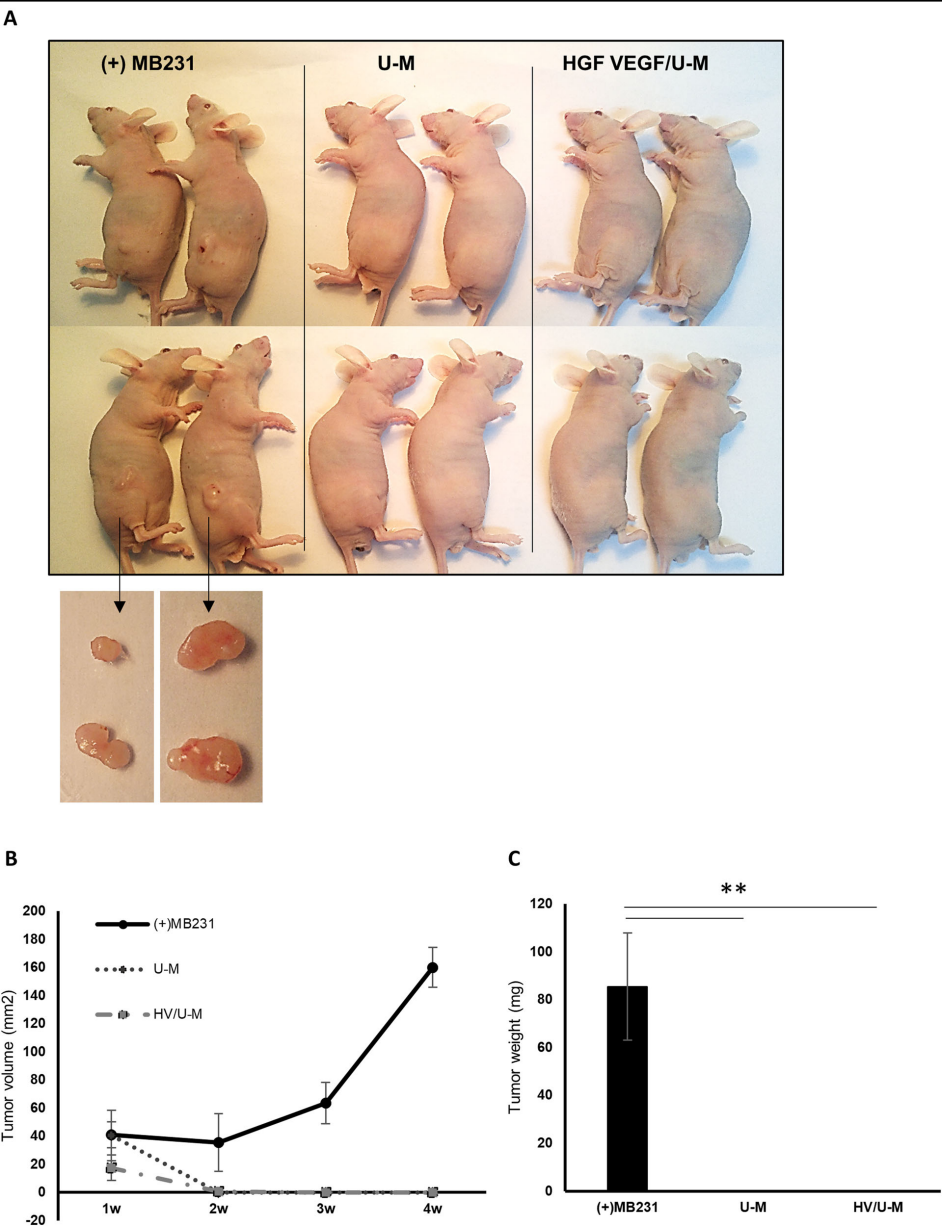
No tumors were formed in nude mice transplanted with the HGF + VEGF/U-Ms. **a** Tumor formation was observed 4 weeks after cell transplantation in BALB/c nude mice. MDA-MB-231 cells were used as a positive control. Tumors did not form in the U-M or HGF + VEGF/U-M groups. **b** The tumor volume was measured weekly. **c** Mice were sacrificed and dissected 4 weeks after transplantation, and tumor formation was analyzed. *N* = 4

We performed a tumor formation assay in vivo to confirm the safety of the functional stem cells. The UCB stem cells that we used secreted VEGF and HGF growth factors; therefore, we hypothesized that these cells may generate cancer cells. Moreover, MSCs exhibit dual potential that can be either pro- or antitumorigenic potential^33^. The transplantation of HGF/U-Ms + VEGF/U-Ms and U-M cells into nude mice produced no tumors up to 30 days post transplantation. However, the MDA-MB-231 breast cancer cell line formed a tumor mass (Fig. 7a, c). The size of the mixture of the HGF/U-Ms + VEGF/U-Ms and Matrigel decreased drastically and disappeared 1–2 weeks post transplantation (Fig. 7b).

## Discussion

Growth factor-secreting stem cells play a pivotal role in stimulating re-endothelialization. These cells serve as carriers of growth factors and sources of endothelial cells. These carrier cells naturally provide HGF and VEGF to other cells, in a similar manner to that of endothelial cells. These cells also release HGF and VEGF in response to doxycycline in a controlled manner. This inducible system reduces the side effects of growth factor overproduction and helps identify optimal conditions for stent-mediated re-endothelialization.

The present study used HGF/U-Ms and VEGF/U-Ms on coronary stents to prevent restenosis and for therapeutic purposes. We also used these HGF- or VEGF-releasing functional stem cells as a source of endothelial cells on the stent for re-endothelialization. MSCs exhibit a high potential to become endothelial cells^31,32^. The present study demonstrated that HGF- or VEGF-releasing MSCs differentiated into endothelial cells and induced tube formation without additional growth factors (Fig. 4a). Angiogenesis and re-endothelialization are distinct processes, but these phenomena share the same regulatory molecular mechanism^33,34^. The improved tube formation confirmed the re-endothelialization of MSCs in vitro. We also demonstrated that the cells transdifferentiated into an endothelial cell layer, or at least provided a suitable microenvironment to form endothelial cells, in the swine model (Fig. 6).

However, some hurdles must be overcome in these cell-based stent systems. Cell attachment to the stent must be improved. A significant number of cells detached from the stent in our experiments due to the physical force exerted on the inner and outer surfaces of the stent during transplantation. Cells remained on the lateral side of the stent, but approximately only one third of the cells remained (Fig. 2b). More cells would enable greater restenosis reduction and re-endothelialization. Future studies should implement better cell attachment technology, and studies to improve cell attachment on the stents are ongoing^35,36^.

In this study, we found the optimal combination of HGF- and VEGF-secreting MSCs for the coronary stent to accomplish optimal restenosis reduction and endothelialization. We tried several different combinations of these two functional MSCs and found that the best combination of HGF- and VEGF-releasing cells was a 5:1 ratio. VEGF produced a significant effect on neointima reduction in the short-term trials (3 days) but not over periods of 2 weeks or longer (Fig. 3). VEGF promotes fibrosis in endothelial cells^16,26,27^. Therefore, longer exposure to a higher amount of VEGF may stimulate fibrosis instead of endothelialization. Conversely, HGF decreased restenosis in the 2-week trials, but this decrease was not sufficient compared to that of the BMS group. The seeding of HGF-secreting MSCs alone on the stent did not reduce restenosis 4 weeks after implantation. Several reports indicated that HGF exclusively stimulated endothelial cell growth and reduced fibrosis without replication of vascular smooth muscle cells^14,29,37–39^. However, the HGF/U-Ms did not survive longer than 2 weeks in vivo, and subsidiary support was necessary. We combined HGF-secreting cells with VEGF-secreting cells to solve this problem. The cross-talk between HGF and VEGF improves stem cell survival and the angioarchitecture and increases cell proliferation and migration^28,34^. This study found the optimal combination of HGF- and VEGF-secreting cells to promote re-endothelialization and minimize the restenosis side effects.

Drug-eluting stents may produce a dramatic reduction in stenosis, and several commercial drug-eluting stents are used clinically^40,41^. However, these stents exhibit the fatal problem of arterial restenosis at later stages. This problem partially occurs due to the inability to form a barrier and protect the artery. Therefore, re-endothelialization is important to protect the coronary artery from restenosis^42^. Another drawback of drug-eluting stents is that the drug itself is antiproliferative and exerts nonspecific effects on the surrounding cells. Therefore, the drug prevents the regeneration of VSMCs and ECs. No other method is available to promote EC regeneration using drug-eluting stents without affecting VSMCs. The functional HGF/VEGF-secreting stem cells seeded on the stent enhanced EC regeneration and prevented VSMC growth.

In summary, coating stents with HGF- and VEGF-secreting mesenchymal stem cells reduced the side effects of coronary stents via the promotion of re-endothelialization. HGF promoted natural endothelialization and produced an even lumen side of the vessel wall. VEGF promoted fibrosis, but it also activated cell survival via the controlled release of the appropriate dose at the appropriate time, which promoted angiogenesis. Our strategy of using growth factor-secreting MSCs has significant implications for clinical stent therapy.

## Supplementary information

Supplementary Information Supplementary Tables 1 and 2, Supplementary Figures 1A, 2B and 3
Supplementary Figure 1
Supplementary Figure 2
Supplementary Figure 3
Supplementary Table 1
Supplementary Table 2
Supplementary Movie 1
