# Supplementary material for: Coronary stents with inducible VEGF/HGF-secreting UCB-MSCs reduced restenosis and increased re-endothelialization in a swine model
Source: Exp Mol Med. 2018 Sep 3;50(9):114. doi: 10.1038/s12276-018-0143-9 (PMC6119684; doi:10.1038/s12276-018-0143-9)
Supplement: Supplementary Information — Supplementary Tables 1 and 2, Supplementary Figures 1A, 2B and 3 [file 12276_2018_143_MOESM1_ESM.docx]

**Supplemental Fig 1.** **Characterization of HGF+VEGF/UCB-MSCs on the stent material. (A)** MSC markers were analyzed by quantitative real-time PCR, and no difference was observed after seeding the cells on stent material. **(B)** Junction PCR of HGF/UCB-MSCs and ∙VEGF/UCB-MSCs confirmed the integration of the HGF and VEGF genes in the human genome.

**Supplemental Fig 2.** **The characteristics of UCB-MSCs are retained after Doxycycline treatment. (A)** The MTT assay exhibited no difference in proliferation after doxycycline treatment of UCB-MSCs. **(B)** Images of cell morphology showed no change after doxycycline treatment 7 days after treatment.

**Supplemental Fig 3. Stents coated with VEGF/UCB-MSCs exhibited reduced neo-intimal area with Doxycycline treatment.** **(A)** OCT results showing neointima degree in the lumen 3 days after transplantation. **(B)** Neointima degree was analyzed by mm^2^ and % of the neointima area.
