# Supplementary figures and images for: Coronary stents with inducible VEGF/HGF-secreting UCB-MSCs reduced restenosis and increased re-endothelialization in a swine model
Source: Exp Mol Med. 2018 Sep 3;50(9):114. doi: 10.1038/s12276-018-0143-9 (PMC6119684; doi:10.1038/s12276-018-0143-9)

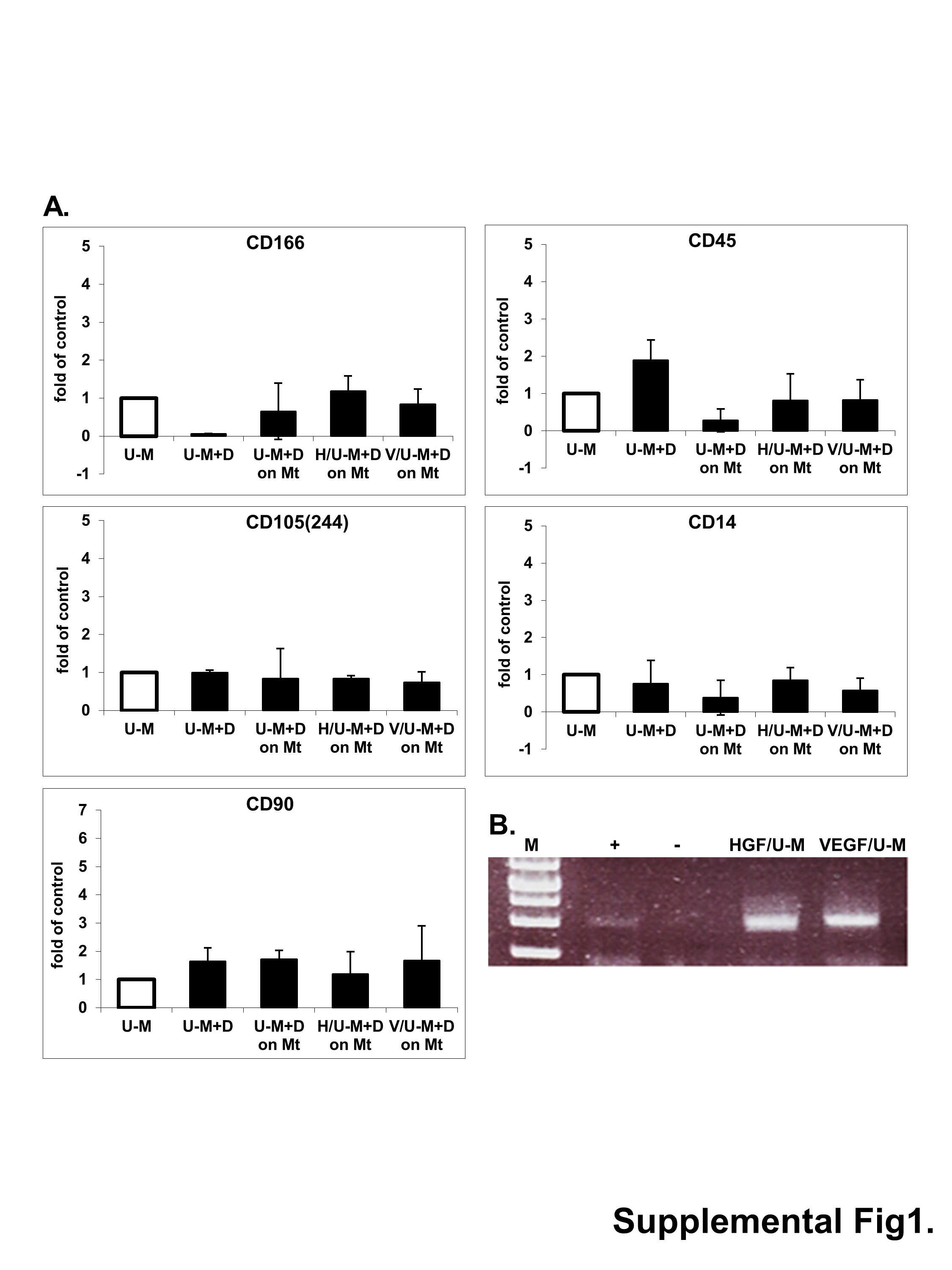

Supplement: Supplementary file 2 — Supplementary Figure 1 [file 12276_2018_143_MOESM2_ESM.tif]

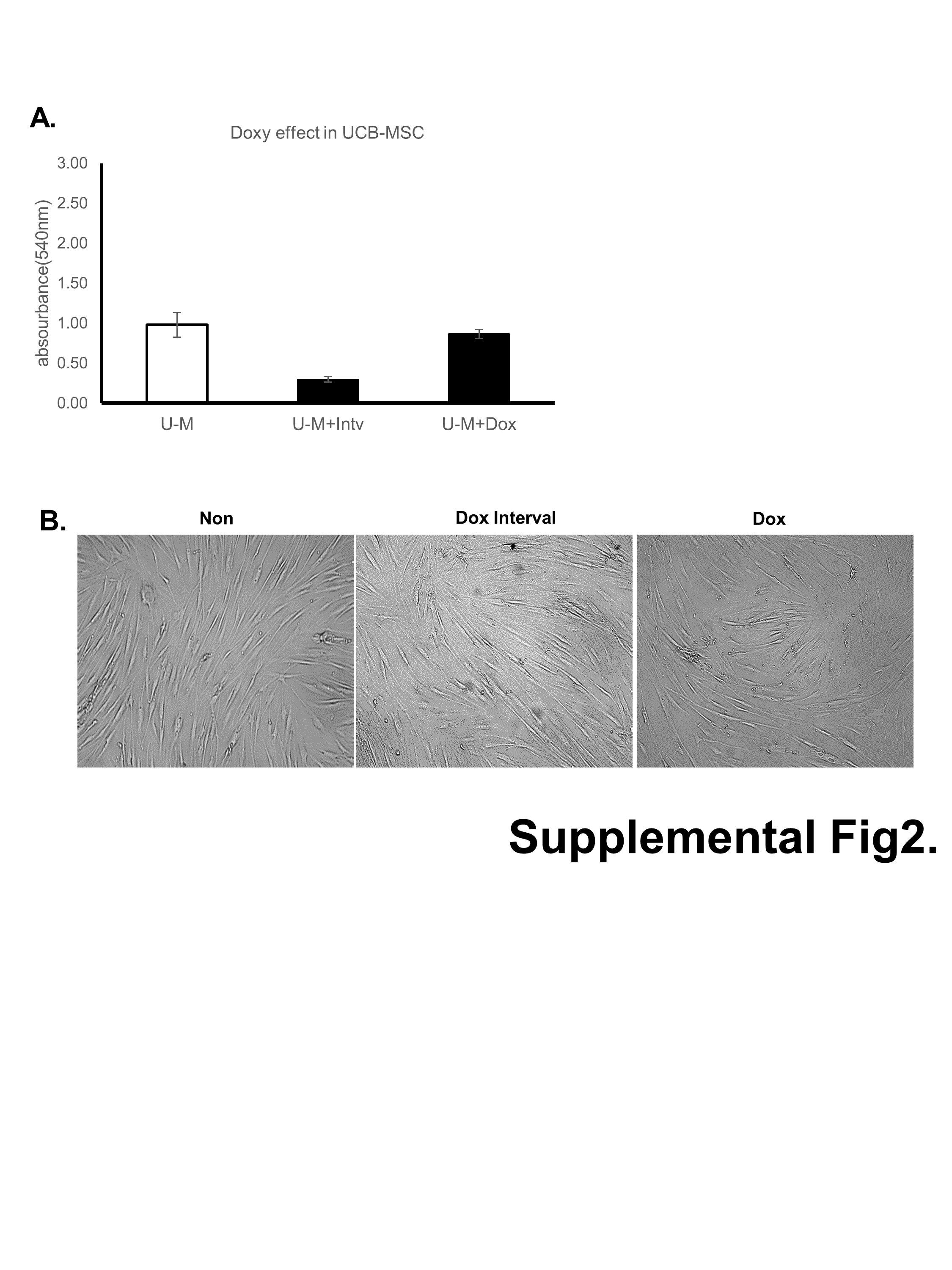

Supplement: Supplementary file 3 — Supplementary Figure 2 [file 12276_2018_143_MOESM3_ESM.tif]

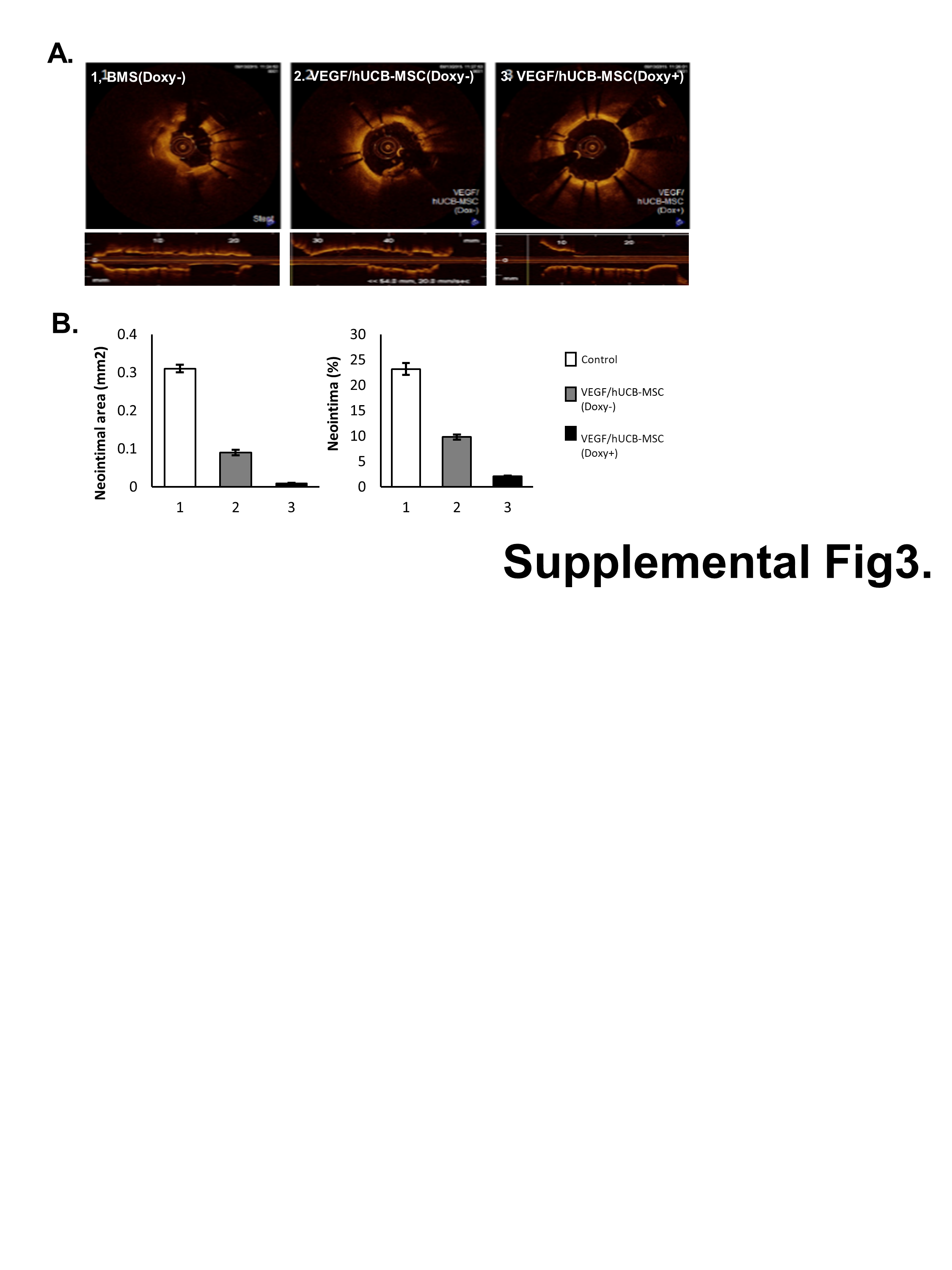

Supplement: Supplementary file 4 — Supplementary Figure 3 [file 12276_2018_143_MOESM4_ESM.tif]

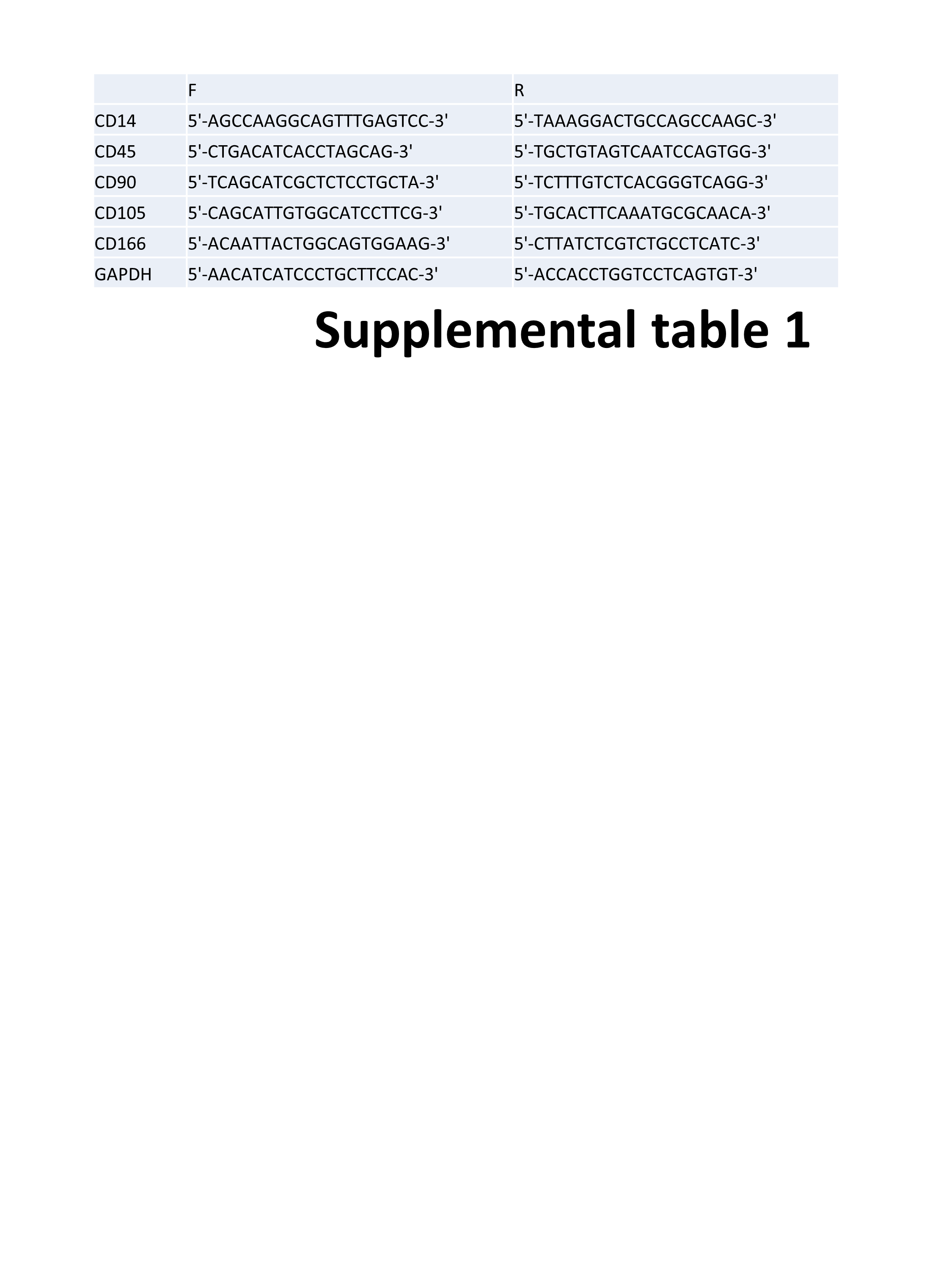

Supplement: Supplementary file 5 — Supplementary Table 1 [file 12276_2018_143_MOESM5_ESM.tif]
